# Habitat segregation between brown bears and gray wolves in a human‐dominated landscape

**DOI:** 10.1002/ece3.4572

**Published:** 2018-11-11

**Authors:** Cyril Milleret, Andrés Ordiz, Guillaume Chapron, Harry Peter Andreassen, Jonas Kindberg, Johan Månsson, Aimee Tallian, Petter Wabakken, Camilla Wikenros, Barbara Zimmermann, Jon E. Swenson, Håkan Sand

**Affiliations:** ^1^ Faculty of Applied Ecology and Agricultural Sciences Inland Norway University of Applied Sciences Koppang Norway; ^2^ Faculty of Environmental Sciences and Natural Resource Management Norwegian University of Life Sciences Ås Norway; ^3^ Grimsӧ Wildlife Research Station Department of Ecology Swedish University of Agricultural Sciences Riddarhyttan Sweden; ^4^ Department of Wildlife, Fish, and Environmental Studies Swedish University of Agricultural Sciences Umeå Sweden; ^5^ Norwegian Institute for Nature Research Trondheim Norway; ^6^ Department of Wildland Resources & Ecology Center Utah State University Logan Utah

**Keywords:** brown bear (*Ursus arctos*), coexistence, competition, gray wolf (*Canis lupus*), habitat segregation, habitat selection

## Abstract

Identifying how sympatric species belonging to the same guild coexist is a major question of community ecology and conservation. Habitat segregation between two species might help reduce the effects of interspecific competition and apex predators are of special interest in this context, because their interactions can have consequences for lower trophic levels. However, habitat segregation between sympatric large carnivores has seldom been studied. Based on monitoring of 53 brown bears (*Ursus arctos*) and seven sympatric adult gray wolves (*Canis lupus*) equipped with GPS collars in Sweden, we analyzed the degree of interspecific segregation in habitat selection within their home ranges in both late winter and spring, when their diets overlap the most. We used the K‐select method, a multivariate approach that relies on the concept of ecological niche, and randomization methods to quantify habitat segregation between bears and wolves. Habitat segregation between bears and wolves was greater than expected by chance. Wolves tended to select for moose occurrence, young forests, and rugged terrain more than bears, which likely reflects the different requirements of an omnivore (bear) and an obligate carnivore (wolf). However, both species generally avoided human‐related habitats during daytime. Disentangling the mechanisms that can drive interspecific interactions at different spatial scales is essential for understanding how sympatric large carnivores occur and coexist in human‐dominated landscapes, and how coexistence may affect lower trophic levels. The individual variation in habitat selection detected in our study may be a relevant mechanism to overcome intraguild competition and facilitate coexistence.

## 1 INTRODUCTION

One of the main objectives in community ecology and conservation is to understand the mechanisms that allow the coexistence of species within the same guild. This understanding requires identifying how sympatric species use limited resources (Armstrong & McGehee, 1976; Chesson, 2000). Indeed, sympatric species sharing similar resources should demonstrate some degree of niche overlap (sensu Hutchinson, 1957), which could lead to interspecific competition (Chesson, 2000; Dufour et al., 2015; Hurlbert, 1978; Lotka, 1925). In turn, interspecific competition can generate differences in habitat selection, which has been observed for various taxa in terrestrial (Holt, 1987) and aquatic realms (Wellborn, Skelly, & Werner, 1996). To buffer competition and allow for coexistence, sympatric species may avoid each other in space and/or time. As niche overlap or niche partitioning is determined by the proximity and abundance of competing species (Hutchinson, 1957; MacArthur & Levins, 1967), long‐term monitoring and analyses of spatial and temporal segregation are important tools for investigating habitat selection patterns in terms of environmental variables and the presence of the other species (Darmon et al., 2012).

Interspecific interactions between species belonging to the same guild, such as apex predators, may influence the population dynamics of species at other trophic levels (Caro & Stoner, 2003; Creel, 2001). Because large carnivores are not suitable for experimental approaches in controlled conditions, studies on the effects of interspecific interactions at the population level are still scarce (Ballard, Carbyn, & Smith, 2003) and they often report on the relationships between dominant and subordinate species (Belant, Griffith, Zhang, Follmann, & Adams, 2010; Darnell, Graf, Somers, Slotow, & Szykman Gunther, 2014). The topic is gaining increasing attention in different ecosystems, thus involving different species in the respective large carnivore guilds (e.g. Elbroch, Lendrum, Allen, & Wittmer, 2015; Périquet, Fritz, & Revilla, 2015). A reasonable approach for identifying the mechanisms allowing carnivore coexistence is to analyze the habitat segregation of carnivores in relation to the habitats they use (Apps, McLellan, & Woods, 2006). Research on fine‐scale spatio‐temporal interactions is needed to advance our understanding of the mechanisms that allow apex predators to coexist and the magnitude of the interspecific interactions between them on lower trophic levels (Linnell & Strand, 2000; Périquet et al., 2015).

**Figure 1:**
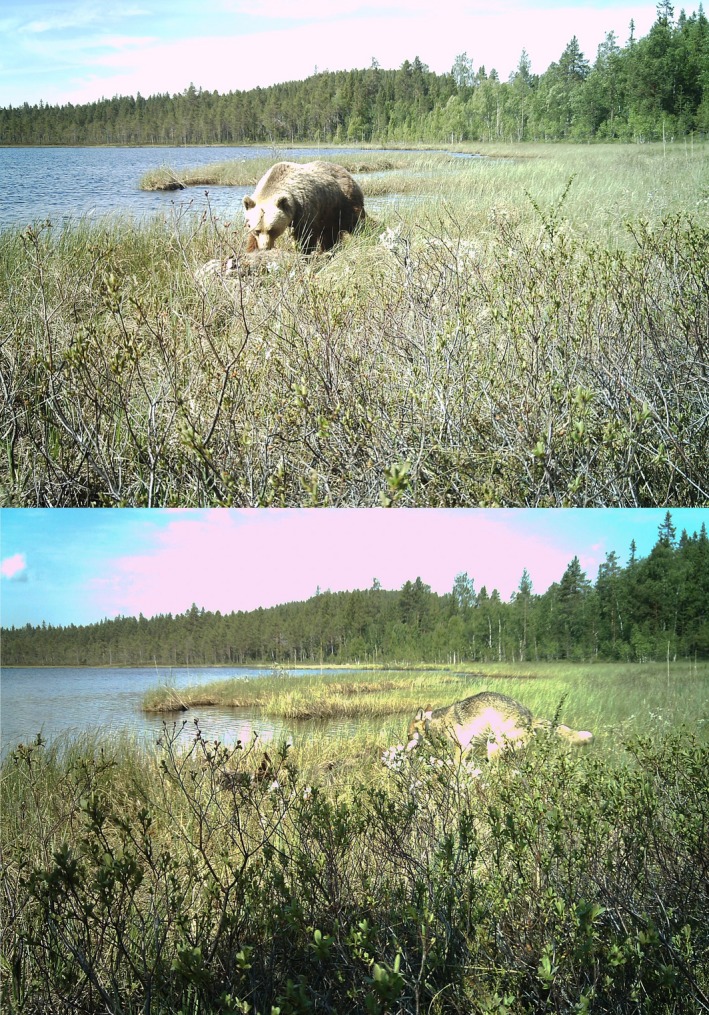
A brown bear (*Ursus arctos*) and a wolf (*Canis lupus*) feeding on the same moose carcass (originally killed by wolves) in southcentral Sweden. ©SKANDULV

Gray wolves (*Canis lupus*) and brown bears (*Ursus arctos*) are two of the largest and most widely distributed apex predators in Eurasia and North America, where they are sympatric in a large part of their ranges (e.g. see IUCN maps; IUCN, 2010; IUCN SSC Bear Specialist Group, IUCN and IBA, 2017; Chapron et al., 2014). Both species are efficient predators of neonate ungulates (Barber‐Meyer, Mech, & White, 2008; Sand et al., 2008; Swenson et al., 2007), and the sharing of this common resource may fuel interspecific competition. In addition, brown bears are efficient scavengers of wolf‐killed ungulates (e.g. Ballard et al., 2003) (Figure 1). Therefore, they are an interesting duo for evaluating the mechanisms involved in the coexistence of apex predators.

Wolves are obligate carnivores, and bears are omnivores. In Scandinavia, moose (*Alces alces*) is the staple prey of wolves (Sand et al., 2008, 2012), whereas the diet of bears includes a wide range of food items (Stenset et al., 2016). Nevertheless, bears are also efficient predators of neonate moose (Dahle et al., 2013; Swenson et al., 2007), and they kleptoparasitize more than half of the wolf kills during spring in central Sweden (Milleret, 2011; Ordiz et al., 2015). Bear density has a negative effect on the probability of wolf pairs establishing in a given area (Ordiz et al., 2015), and wolf kill rates are lower when bears are present (Tallian et al., 2017). However, no effects of wolves have ever been documented on brown bears at the population level. These findings suggest that wolves and bears may display consumer–resource interactions, such as parasitism, where bears benefit and wolves lose as a result of their interaction (schematically, bear: +, wolf: −), and exploitative competition, where both bears and wolves would lose as a result of the interaction (bear: −; wolf: −).

In order to obtain a comprehensive understanding of the mechanisms that allow the coexistence of free‐ranging apex predators, it is important to understand how predators select habitat in shared landscapes. Indeed, the spatial effects of biotic interactions on species distributions have rarely been investigated (Araújo & Rozenfeld, 2014). Two species that select similar resources may never interact directly, and the spatial scale at which competition becomes visible depends on the nature and strength of the interactions. According to Araújo and Rozenfeld (2014), the effects of parasitism should be visible across all spatial scales, if one species has strong positive benefits on the other, but the effects of competition should only be visible at fine spatial scales. Although wolves and bears are sympatric within similar habitat types at the landscape level (May et al., 2008; Ordiz et al., 2015), segregation could occur at finer spatio‐temporal scales, for example, within different habitats in their home ranges or at very fine scale, for example, at feeding sites. However, we know of no studies that have examined whether the selection of different habitats within home ranges, that is, habitat segregation, could be used by wolves and bears as a mechanism of coexistence.

We used GPS locations from sympatric radio‐marked wolves and bears to quantify their habitat segregation in central Sweden. The effect of bears on wolves (i.e. parasitism of wolf kills and exploitative competition of common prey, i.e. neonate moose) may cause wolves to segregate from bears. Because wolves are strictly carnivorous and bears omnivorous, they may express different habitat selection patterns. Therefore, we hypothesized the existence of habitat segregation that was larger than expected by chance between wolves and bears. We focused our analysis in late winter (when bears come out of winter dens) and spring (i.e. the period when both wolves and bears prey on just born moose), which may lead to higher trophic overlap than during the rest of year, thus helping us to infer the degree of interaction between wolves and bears. We used habitat‐, prey‐, and human‐related variables to quantify the habitat selection of wolves and bears, because of the documented influence of these factors on wolf and bear distribution and behavior (e.g. Ordiz, Kindberg, Sæbø, Swenson, & Støen, 2014; Ordiz et al., 2015; Zimmermann, Nelson, Wabakken, Sand, & Liberg, 2014). Then, we quantified habitat segregation between wolves and bears using a multivariate approach based on the niche concept. Because of the above‐mentioned negative effects of bear density on the probability of establishment by wolf pairs (Ordiz et al., 2015) and the kleptoparasitism of wolf kills by bears in Scandinavia and elsewhere (Tallian et al., 2017), we hypothesize that wolves and bears will segregate more than expected by chance. Our study may advance current knowledge of the ecological mechanisms that drive interspecific interactions between apex predators and allow their coexistence in human‐dominated landscapes.

## 2 MATERIALS AND METHODS

### 2.1 Study area

**Figure 2:**
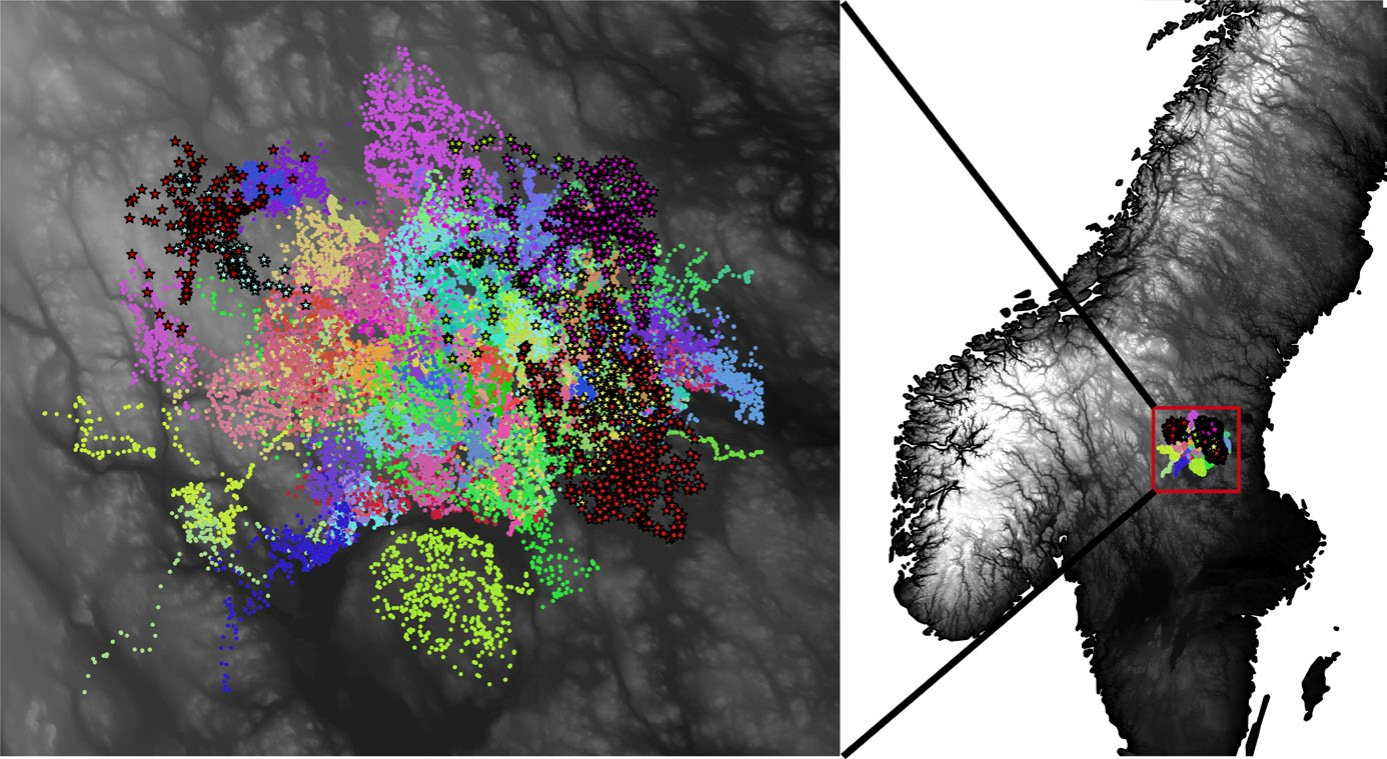
Map of the study area in central Sweden. The elevational gradient is shaded from black (low elevation) to white (high elevation). GPS locations from brown bears (circles) and gray wolves (stars with black outline) are shown in different colors for each individual during the study period (2010–2015)

Our study area was located in central Sweden (Figure 2; elevation: 100–830 m), mainly composed of boreal forest, with the coniferous species Scots pine (*Pinus sylvestris)* and Norway spruce (*Picea abies*) covering ~60% of the area (Supporting Information Table S1). Human density was low, with 1–7 inhabitants/km^2^ in 2012 (http://www.scb.se), but logging is very intense and therefore the landscape is crisscrossed by many roads (1 ± 0.5 km/km^2^; Ordiz et al., 2014), which are also used for a variety of other human activities, including moose and bear harvest in the fall. Snow usually covers the ground from December to March. Bear density approached 30 bears/1,000 km^2^ (Solberg, Bellemain, Drageset, Taberlet, & Swenson, 2006). The first two wolf territories established within the study area were detected during the winter 2000/2001 (Wabakken, Aronson, Sand, Steinset, & Kojola, 2002). Since then, one to eight territories have been recorded annually during systematic snow‐tracking surveys (e.g. Liberg et al., 2012; Wabakken, Svensson, Maartmann, Åkesson, & Flagstad, 2016). Two more members of the large carnivore guild were present in the study area, Eurasian lynx (*Lynx lynx*) and wolverine (*Gulo gulo*). Moose was the most abundant ungulate prey species, with average density estimates of 0.7–1.6/km^2^; the only alternative ungulate was roe deer (*Capreolus capreolus*), with a very low estimated density of 0.05–0.08/km^2^ (Sand, Eklund, Zimmermann, Wikenros, & Wabakken, 2016).

### 2.2 Study animals

Wolves and bears were captured following ethically approved veterinary procedures described in Arnemo, Evans, and Fahlman (2012) and were equipped with GPS–GSM neck collars (VECTRONIC Aerospace GmbH, Berlin, Germany). At least one territory‐holding, scent‐marking adult wolf per breeding pair was collared in the three known wolf territories in the core of our study area (Kukumäki, Tandsjön, and Tenskog territories). Wolf collars recorded positions at 60‐min intervals throughout the study periods. Because wolf pairs spend most of their time together outside the reproduction period (Zimmermann, Sand, Wabakken, Liberg, & Andreassen, 2015), we only retained GPS data from one of the pair members for the analysis (see study period paragraph for more details). We used data from 53 radio‐collared bears, whose collars were programmed to record locations every hour during our study periods. About 80% of the adult female bears and 50% of the adult male bears in the study area are radio‐collared (Bellemain, Swenson, & Taberlet, 2006). During 1 January 2010–31 December 2014, we obtained 931,277 GPS locations from 79 bear‐years and 25,709 GPS locations from seven wolf territory‐years (Figure 2, Supporting Information Table S4).

### 2.3 Landscape characteristics

We characterized land cover into twelve categories (25‐m resolution), according to the “Svenska Marktäckedata” (SMD) land cover map (Lantmäteriet, Sweden; Supporting Information Table S1) constructed from satellite images taken on 12 September 2002. Because of intensive logging in the study area, we updated the vegetation map using information about forest clear‐cuts (Nicholson, Milleret, Månsson, & Sand, 2014) performed between 12 September 2002 and 1 January 2012 (mid‐date of the study period). We obtained this information from the Swedish Forestry Agency (http://www.skogsstyrelsen.se). To account for succession of the vegetation, the classes *Clearcuts* and *Young Forest* in 2002 were reconsidered as *Young Forest* and *Mid‐age coniferous forest* (Supporting Information Table S1), respectively, in the updated map (Nicholson et al., 2014).

We also computed distances in km from main (paved) and secondary (gravel) roads. Finally, we used a digital elevation model (GSD‐Elevation data, Grid 2+; http://www.lantmateriet.se) to extract elevation and calculate a terrain ruggedness index (TRI; Sappington, Longshore, & Thompson, 2007). We computed three different TRIs using moving windows of different sizes (3 × 3; 5 × 5; 7 × 7) with a cell resolution of 10 m. A preliminary analysis showed that TRI7 (7 × 7) was better at explaining wolf and bear habitat selection (i.e. higher contribution on the axes of the K‐select), and it was therefore retained for the subsequent analyses.

### 2.4 Moose occurrence

Moose is the main ungulate prey of wolves and bears in Scandinavia (Tallian et al., 2017), and most documented wolf–bear interactions occur near kill sites (Ballard et al., 2003). We used moose pellet counts (Neff, 1968) to compute a moose resource selection function that predicted moose occurrence within the study area. Pellet counts can be used to document moose habitat selection during winter and early spring (Månsson, Andrén, & Sand, 2011), and they are the best available data describing moose habitat selection patterns in our study area. We conducted pellet count surveys during spring in each wolf territory: Tenskog (2010; 1,960 sample plots); Tandsjön (2012; 2,600 plots); and Kukumäki (2014; 1,920 plots). The circular sample plots of 100 m^2^ were placed along the 1 × 1 km squares that were systematically distributed within the 100% minimum convex polygon (MCP) of the wolf territory (Zimmermann et al., 2015). Each square boundary contained 40 sample plots. We searched for moose pellets and determined their age based on their structure, consistency, color, and position in relation to the vegetation, in order to count only pellet groups produced after leaf fall of the previous autumn (Gervasi et al., 2013; Rönnegård, Sand, Andren, Månsson, & Pehrson, 2008).

Based on the pellet survey results, we computed a resource selection function (Manly, ‎McDonald, Thomas, ‎McDonald, & Erickson, 2002) with the number of pellets counted in each plot as the response variable (Gervasi et al., 2013). We used all land‐use descriptors as explanatory variables (except the variable “Water”) and calculated the proportion of land‐use characteristics (Supporting Information Table S1) using a moving window (5 × 5 cells; 25 × 25 m cell size). Due to the high number of zeros in the data, we applied a zero‐inflated negative binomial model (Zuur, Ieno, Walker, Saveliev, & Smith, 2009). We started from a fully parameterized model and used Akaike information criterion (AIC) to select the most parsimonious model. Models with a ∆AIC < 2 were considered equally supported by the data.

We performed a collinearity analysis and detected no excessive level of correlation in the set of explanatory variables (all Pearson's *r* < 0.3). We used k‐fold cross‐validation to evaluate model performance (Boyce, Vernier, Nielsen, & Schmiegelow, 2002). Performance never exceeded 30% (Spearman's *r*), which is in accordance with other studies using similar data and models (e.g. Bouyer et al., 2015). To predict spatial variation of moose occurrence within our study area, we performed model averaging to obtain coefficients for the variables retained in the best models, using the “MuMIn” package in R (Barton, 2009). The predicted values were used as a relative index of moose occurrence (*Moose_pred*) during winter–spring in the subsequent analyses (See Supporting Information Tables S2 and S3, and Supporting Information Figure S1 for further information).

### 2.5 Study periods

**Figure 3:**
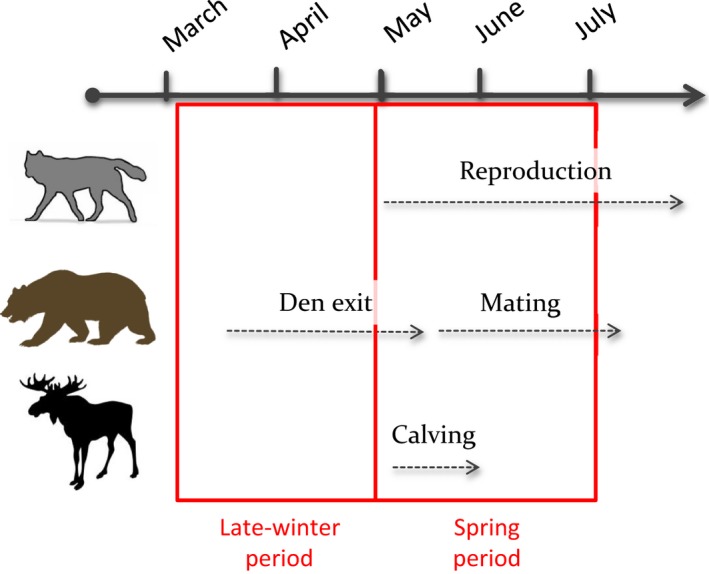
Biological justification of two study periods (red full boxes) in late winter (1 March–30 April) and spring (1 May–30 June) to analyze habitat selection of gray wolves and brown bears in central Sweden. Dashed gray lines illustrate the approximate duration of specific behaviors of wolves, bears, and moose, the main ungulate prey species for both carnivores

We defined two study periods in late winter and spring to take into account the marked seasonal variation in the annual cycle of wolves and bears (Figure 3), because seasonality is an important factor to consider in studies of interspecific interactions (e.g. Basille, Fortin, Dussault, Ouellet, & Courtois, 2013; Bastille‐Rousseau et al., 2016). During the late‐winter period (1 March–30 April), male bears start to leave their winter dens (Manchi & Swenson, 2005). The spring period (1 May–30 June) overlaps with wolf reproduction (Alfredéen, 2006; Nonaka, 2011; Mech & Boitani, 2010) and the bear mating season (Dahle & Swenson, 2003). The latter period also includes the birth of moose calves (Markgren, 1969), which are a highly utilized prey by both wolves and bears in several ecosystems, including our studied ecosystem (Rauset, Kindberg, & Swenson, 2012; Brockman, Collins, Welker, Spalinger, & Dale, 2017, Tallian et al., 2017). During the late‐winter period, we only used GPS locations from one member of the pair (male or female, Supporting Information Table S4), because both pair members usually travel together outside the reproduction period (Peterson, Jacobs, Drummer, Mech, & Smith, 2002; Zimmermann et al., 2015). During the spring period, we used GPS locations from the male wolves, except for one territory‐year, when only the collar of the female was functioning (Supporting Information Table S4). We prioritized locations from the males over females in the spring period, because the females are more stationary near the den during pup rearing (Alfredéen, 2006). Wolf pairs reproduced in all years except one (“Tenskog 2010,” Supporting Information Table S4).

### 2.6 Habitat selection

We quantified habitat selection by wolves and bears within their home range (third order of selection; Johnson, 1980). To quantify habitat segregation between the species, we used a multivariate approach that relies on the concept of ecological niche, K‐select (Calenge, Dufour, & Maillard, 2005; Darmon et al., 2012). Each habitat variable defines one dimension in the ecological space, and the vector (marginality) of the differences between average available and used habitat quantifies the strength and direction of the selection (Calenge et al., 2005). Therefore, the direction (positive or negative) indicates habitats used, and the marginality “score” indicates the strength of the use. Average conditions were defined using a 95% MCP for each individual‐year. In order to extract the relevant aspects of habitat selection, we computed a principal component analysis from the marginality vectors. For further details on mathematical procedures of K‐select, see Calenge et al. (2005). Using linear mixed models (Bates, Mächler, Bolker, & Walker, 2015), we then tested whether species, bear reproductive status, time of the day, or interactions among these variables could explain differences in the centered marginality values obtained on each axis of the K‐select. We included individual identity as a random intercept to account for individual heterogeneity and repeated measures of its habitat selection. We then selected the most parsimonious models fitted with the maximum likelihood method (Zuur et al., 2009) using AIC, and, when the ∆AIC between competing models was <2, we retained the simplest model (Burnham & Anderson, 2002).

To account for individual variability in habitat selection (Leclerc et al., 2015; Uboni, Smith, Mao, Stahler, & Vucetich, 2015), individuals that were monitored in multiple years were considered as different individuals in each year in the K‐select analysis. Because wolves and bears are mostly active from dusk to dawn (Ordiz et al., 2014; Sand, Zimmermann, Wabakken, Andrèn, & Pedersen, 2005), we also separated habitat selection for each individual into day and night, using monthly sunset and sunrise tables. However, we defined the 95% MCP using all GPS locations from both day and night as available for each individual‐year. In brown bears, behavior varies markedly due to sex and reproductive status, for example, in terms of daily movement patterns (Ordiz et al., 2014) and habitat selection (Steyaert, Kindberg, Swenson, & Zedrosser, 2013). Therefore, we distinguished habitat selection and segregation among the following bear classes: females with dependent offspring; single females; adult males; and subadult (<4 years old) bears of both sexes. We did not make any such classes for wolves, because of the low sample size of different categories. The different available habitats among home ranges of different individual bears and wolves could lead to functional responses (Mysterud & Ims, 1998). However, we could not detect any functional response (Supporting Information Figure S2).

### 2.7 Habitat niche segregation

**Figure 4:**
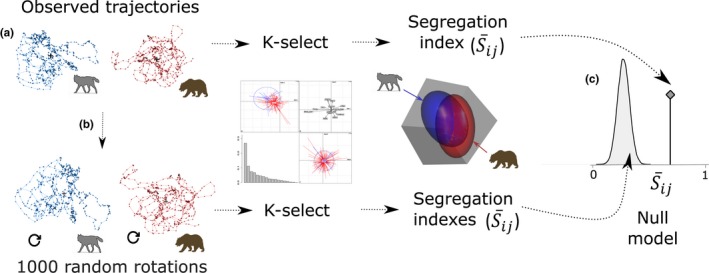
Flowchart illustrating the procedure to analyze gray wolf and brown bear habitat selection and segregation in central Sweden. (a) Observed trajectories from each individual wolf and bear were used to quantify habitat selection with the K‐select (See Section 2.6). The four plots illustrate results obtained with the K‐select (Supporting Information Figures S3 and S4) which we used to calculate a segregation index (${\overset{¯}{S}}_{\mathrm{ij}}$ = 0–100%) in terms of habitat selected by wolves and bears. This segregation index was calculated over all axes of the K‐select (i.e. weighted by the respective eigenvalues obtained on each axis). The 3D plot illustrates the habitat niche (ellipses) of wolves (blue) and bears (red) on only three different axes for illustrative purposes (${\overset{¯}{S}}_{\mathrm{ij}}$ was actually performed on 18 different axes identified by the K‐select). The area of overlap between the two ellipses illustrates the area of overlap between wolves and bears, whereas the area outside represents habitat segregation. (b) To create random use of the habitat by both species, we randomly rotated the complete trajectory from each individual around its centroid 1,000 times. The same procedure described in (a) was used for each of the 1,000 simulated datasets. (c) The 1,000 segregation indexes were used to create the null model (density distribution curve: null hypothesis), the random distribution of the segregation index under random habitat used by both species. If the observed segregation index (vertical line, at the left of the density distribution curve) was ≥95% of the simulated segregation indexes, we rejected our null hypothesis and accepted our alternative hypothesis that segregation between both species was higher than expected by chance

In order to quantify the degree of segregation between bears and wolves, we conducted an ad hoc analysis, based on scores of the locations obtained for each individual, and on each dimension of the ecological space of the K‐select (Figure 4). For the scores of each individual on each dimension, we computed a nonparametric Gaussian kernel density estimation (Geange, Pledger, Burns, & Shima, 2011) using the “rule of thumb” to obtain the bandwidth value (Silverman, 1986). We then paired each kernel estimate with each individual/species and calculated the area that did not overlap (Segregation: $S_{ij_{e}}$) between the two distributions of species *i* and *j* for each axis *e*, using Equation (1), where *f*(*x*) and *g*(*x*) are the probability density function of species *i* and *j*. Finally, we computed an overall segregation index ${\overset{¯}{S}}_{\mathrm{ij}}$ weighed by the eigenvalues (*λ*) of each dimension *e* of the K‐select (Equation (2)). The index ($S_{\mathrm{ij}}$) ranged between 1 (complete segregation) and 0 (no segregation) between species *i* and *j*.

(1) $$S_{ij_{e}}=\int \text{min}\left[f(x)-g(x)\right]dx$$

(2) $${\overset{¯}{S}}_{\mathrm{ij}}=\frac{\sum_{e}^{n}S_{ij_{e}}\lambda_{e}}{\sum_{e}^{n}\lambda_{e}}.$$

To test whether or not wolves and bears segregated more than expected by chance, we computed null models describing habitat selection of individuals from both species under random use of the habitats (Figure 4). We followed the methodology used by Martin, Calenge, Quenette, and Allainé (2008) to simulate random habitat selection, by randomly rotating the complete trajectory of each individual bear and wolf around the centroid of their respective observed trajectories 1000 times. We then computed the K‐select and the overall segregation index (${\overset{¯}{S}}_{\mathrm{ij}}$) as described above for each of the 1,000 simulated datasets. We used the 1,000 ${\overset{¯}{S}}_{\mathrm{ij}}$ values to build a null distribution of habitat segregation between wolves and bears under random habitat selection. We then used randomization procedures to compare observed indices ($S_{ij_{e}}$) with the null distribution of segregating indexes obtained from the simulated datasets. We calculated *p*‐values as the proportion of simulated segregation indexes that were superior or equal to the observed segregation index. A *p*‐value <0.05 was used to reject the null hypothesis that there was no habitat segregation between wolves and bears and accept our alternative hypothesis that wolves and bears segregated more than expected by chance. All analyses were conducted using R 3.3.1 (R Core Team, 2015) and package “adehabitat” (Calenge, 2006).

## 3 RESULTS

### 3.1 Late winter (1 March–30 April)

**Figure 5:**
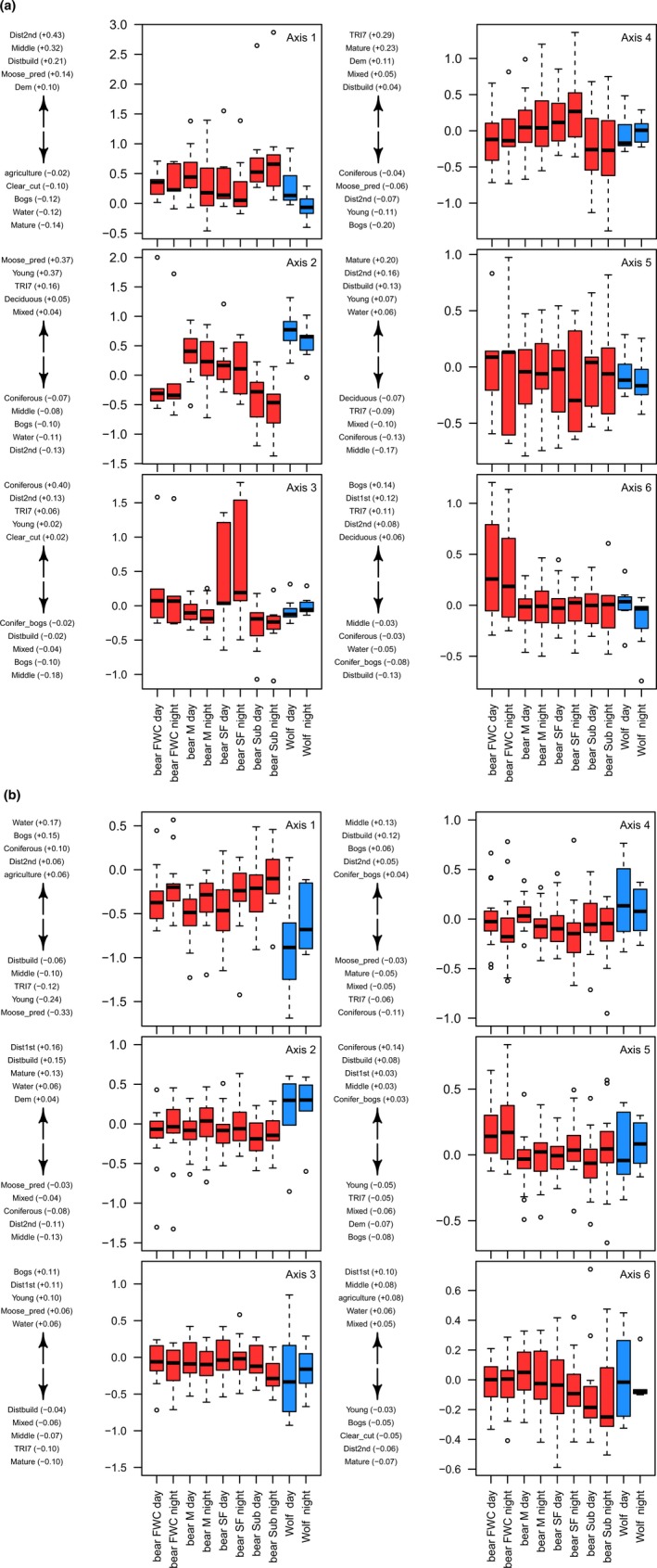
Box plot of the K‐select analysis for habitat selection of gray wolves (blue) and brown bears (red) in central Sweden for the periods, (a) late‐winter period (1 March–30 April) and (b) spring (1 May–30 June). Box plots show marginality scores per species and reproductive status for axes 1–6 of the K‐select, respectively. The five variables contributing the most on each axis are shown on the left side of each box plot, with positive values above the arrow and negative values below the arrow. The scores of the five variables contributing the most are represented in brackets

The first six axes of the K‐select explained 83% of the marginality and were retained for the analysis (Figure 5a). Wolves and bears segregated from each other more than expected by chance (${\overset{¯}{S}}_{\mathrm{ij}}$ = 14%, *p* ≤ 0.01). Specifically, wolves segregated more than expected from male bears ( ${\overset{¯}{S}}_{\mathrm{ij}}$ = 14.1%, *p* ≤ 0.01) and females with cubs (${\overset{¯}{S}}_{\mathrm{ij}}$ = 27.7%, *p* ≤ 0.05) during the night (Table 1). Species and time of day were important variables explaining variation in marginality scores on different axes (Table 2A). Wolves tended to select for moose occurrence, young forests, and rugged terrain more than bears did (Figure 5a, Axis 2), as shown by the negative beta values for all bear classes (Table 3A, Axis 2). Nevertheless, we also observed similarities in habitat selection among wolves and bears. Both species tended to select mid‐age forests and areas farther away from secondary roads and buildings during the day compared to the night. For the axes 4, 5, and 6, individual variability in habitat selection was not explained by species‐specific or intraspecific (i.e. reproductive status) characteristics (Figure 5a).

**Table 1:** Paired comparisons of weighted habitat niche segregation (${\overset{¯}{S}}_{\mathrm{ij}}$) in percentages between gray wolves and brown bears in Sweden, 2010–2015

	Day FWC	Day M	Day SF	Day Sub	Day Wolf	Night FWC	Night M	Night SF	Night Sub	Night Wolf
Day FWC		11.1	9.1	12.1	23.8[Link]	4.7[Link]	8.6	10.3	12.2	16.5[Link]
Day M	24.2		9.0	9.0	21.8[Link]	12.1[Link]	6.4[Link]	12.5[Link]	11.8[Link]	13.1
Day SF	25.7	18.2		10.3	12.4[Link]	9.6	7.7	5.4[Link]	12.2	13.9
Day Sub	27.3	18.1	23.7		24.5[Link]	12.7	8.5	11.7	6.6[Link]	15.9[Link]
Day Wolf	26.7	12.2	16.6	21.8		24.9[Link]	22.6[Link]	25.1[Link]	26.4[Link]	11.7[Link]
Night FWC	5.9	23.5	24.8	25.8	26.0		9.1	8.9	11.2	17.0[Link]
Night M	24.9	6.1[Link]	19.7	19.1	13.1[Link]	24.0		9.5	9.7	14.0[Link]
Night SF	26.9	18.6	6.4[Link]	24.9	17.6	25.9	19.4		11.4	16.0[Link]
Night Sub	27.8	21.1	25.0	5.2	24.0	26.2	21.7	26.4		17.6[Link]
Night Wolf	28.7[Link]	15.5[Link]	18.3	24.2	8.2[Link]	27.7[Link]	14.1[Link]	18.5	26.1	

Notes

Segregation indexes for the late‐winter period (1 March–30 April) are shown on the lower diagonal (i.e. the cells shaded in black) of the table, and the upper diagonal (i.e. the cells shaded in black) corresponds to the spring period (1 May–30 June). Indexes of segregation between wolves and brown bears are shaded in gray, and unshaded indexes show intraspecific indexes of segregation. The segregation indexes in bold show that segregation was significantly larger than expected by chance.

FWC: bear females with cubs; M: male bear; SF: single female bear; Sub: subadult bear.

The superscripts on the right side of the indexes show the degree of significance:

*p* value ≤ 0.05.

*p* value ≤ 0.01.

*p* value ≤ 0.001.

**Table 2:** AIC model selection results for the marginality scores of the K‐select for each axis and each study period in Sweden, (A) late‐winter: 1 March–30 April; and (B) spring: 1 May–30 June, with the variables Time (day/night), Species (gray wolf/brown bear), and reproductive status, that is, “Repro” (bear females with cubs, single bear females, adult bear males, subadult bears, and wolf)

(A) Late‐winter period						(B) Spring period					
Model	K	AIC	Delta_AIC	AICWt	LL	Model	K	AIC	Delta_AIC	AICWt	LL
Axis 1						Axis 1					
Time	4	93.15	0	0.51	−42.58	Time × Repro	12	82.06	0.00	0.92	−29.03
Species × Time	6	93.60	0.45	0.41	−40.80	Species × Time	6	87.38	5.32	0.06	−37.69
Null	3	98.63	5.48	0.03	−46.31	Time	4	90.08	8.02	0.02	−41.04
Time × Repro	12	99.16	6.01	0.03	−37.58	Repro	7	101.12	19.06	0.00	−43.56
Species	4	99.64	6.49	0.02	−45.82	Species	4	109.19	27.13	0.00	−50.60
Repro	7	102.17	9.02	0.01	−44.08	Null	3	113.93	31.86	0.00	−53.96
Axis 2						Axis 2					
Repro	7	107.30	0	0.88	−46.65	Time	4	33.45	0.00	0.49	−12.73
Time × Repro	12	111.23	3.93	0.12	−43.62	Species × Time	6	34.46	1.01	0.29	−11.23
Time	4	129.56	22.26	0	−60.78	Species	4	36.30	2.84	0.12	−14.15
Species	4	129.79	22.49	0	−60.90	Null	3	37.30	3.84	0.07	−15.65
Species × Time	6	129.99	22.69	0	−58.99	Repro	7	39.23	5.77	0.03	−12.61
Null	3	131.23	23.93	0	−62.62	Time × Repro	12	42.97	9.51	0.00	−9.48
Axis 3						Axis 3					
Repro	7	60.14	0	0.67	−23.07	Time × Repro	12	6.01	0.00	0.42	9.00
Time × Repro	12	61.59	1.44	0.33	−18.79	Time	4	6.43	0.43	0.34	0.78
Null	3	80.49	20.35	0	−37.25	Species × Time	6	8.30	2.29	0.13	1.85
Time	4	82.10	21.95	0	−37.05	Repro	7	9.13	3.13	0.09	2.43
Species	4	82.26	22.11	0	−37.13	Null	3	12.22	6.21	0.02	−3.11
Species × Time	6	85.71	25.57	0	−36.86	Species	4	13.72	7.71	0.01	−2.86
Axis 4						Axis 4					
Repro	7	106.48	0.00	0.38	−46.24	Time	4	−5.39	0.00	0.77	6.70
Null	3	106.77	0.29	0.33	−50.38	Species × Time	6	−2.97	2.42	0.23	7.49
Time	4	108.50	2.02	0.14	−50.25	Null	3	5.82	11.21	0.00	0.09
Species	4	108.75	2.28	0.12	−50.38	Species	4	6.21	11.60	0.00	0.89
Species × Time	6	112.48	6.00	0.02	−50.24	Time × Repro	12	7.17	12.56	0.00	8.42
Time × Repro	12	115.50	9.02	0.00	−45.75	Repro	7	11.45	16.84	0.00	1.27
Axis 5						Axis 5					
Null	3	55.88	0.00	0.46	−24.94	Repro	7	−67.54	0.00	0.50	40.77
Species	4	57.87	1.98	0.17	−24.93	Time	4	−66.25	1.29	0.26	37.13
Time	4	57.88	2.00	0.17	−24.94	Time × Repro	12	−65.29	2.25	0.16	44.64
Repro	7	57.92	2.03	0.17	−21.96	Species × Time	6	−62.30	5.24	0.04	37.15
Species × Time	6	61.56	5.67	0.03	−24.78	Null	3	−62.15	5.39	0.03	34.08
Time × Repro	12	65.51	9.63	0.00	−20.76	Species	4	−60.20	7.34	0.01	34.10
Axis 6						Axis 6					
Repro	7	1.04	0.00	0.28	6.48	Null	3	−64.94	0	0.44	35.47
Null	3	1.27	0.23	0.25	2.37	Time	4	−64.00	0.94	0.28	36.00
Time	4	2.14	1.10	0.16	2.93	Species	4	−63.02	1.92	0.17	35.51
Species × Time	6	2.15	1.10	0.16	4.93	Repro	7	−61.32	3.62	0.07	37.66
Species	4	2.87	1.83	0.11	2.56	Species × Time	6	−60.13	4.81	0.04	36.06
Time × Repro	12	5.47	4.43	0.03	9.26	Time × Repro	12	−52.94	11.99	0	38.47

*Notes*

Number of parameters (K), Akaike information criteria (AIC), ∆AIC, AIC weight (AICWt), and log likelihood (LL).

**Table 3:** Parameter estimates for each of the fixed effects retained in the best linear mixed models to test whether species (gray wolf and brown bear), bear reproductive status, time of the day, and or interactions among these variables could explain differences in the centered marginality values obtained on each axis of the K‐select, based on AIC model comparison (Table 2)

(A) Late‐winter period					(B) Spring period				
	Beta	*SE*	LCI	UCI		Beta	*SE*	LCI	UCI
Axis 1					Axis 1				
Intercept[Link]	0.51	0.10	0.30	0.71	Intercept[Link]	−0.86	0.16	−1.19	−0.54
Night	−0.15	0.05	−0.26	−0.04	Wolf_night	0.28	0.14	0.00	0.56
					Bear_FWC_day	0.57	0.19	0.20	0.94
Axis 2					Bear_FWC_night	0.74	0.19	0.37	1.11
Intercept[Link]	0.64	0.28	0.07	1.21	Bear_M_day	0.28	0.18	−0.08	0.64
Bear_FWC	−0.58	0.37	−1.32	0.17	Bear_M_night	0.50	0.18	0.15	0.86
Bear_M	−0.36	0.32	−0.99	0.28	Bear_SF_day	0.34	0.18	−0.02	0.69
Bear_SF	−0.38	0.32	−1.03	0.27	Bear_SF_night	0.55	0.18	0.20	0.91
Bear_Sub	−1.20	0.32	−1.85	−0.56	Bear_Sub_day	0.59	0.18	0.24	0.95
Axis 3					Bear_Sub_night	0.76	0.18	0.40	1.12
Intercept[Link]	−0.05	0.37	−0.79	0.69					
Bear_FWC	0.32	0.47	−0.64	1.28	Axis 2				
Bear_M	0.40	0.40	−0.40	1.21	Intercept[Link]	−0.10	0.04	−0.17	−0.02
Bear_SF	0.11	0.41	−0.70	0.93	Night	0.08	0.03	0.01	0.14
Bear_Sub	−0.36	0.40	−1.17	0.44					
					Axis 3				
Axis 4					Intercept[Link]	−0.10	0.04	−0.17	−0.02
Intercept	0.26	0.08	0.26	0.52	Night	0.08	0.03	0.01	0.14
									
Axis 5					Axis 4				
Intercept	0.36	0.23	0.19	0.27	Intercept[Link]	0.00	0.04	−0.07	0.08
					Night	−0.10	0.03	−0.15	−0.05
Axis 6									
Intercept	−0.01	0.06	0.13	0.19	Axis 5				
					Intercept[Link]	0.00	0.03	−0.06	0.06
					Night	0.06	0.02	0.01	0.10
					Axis 6				
					Intercept	0.03	0.03	−0.08	0.02

*Notes*

Estimates are presented for each study period, (A) late‐winter: 1 March‐30 April; and (B) spring: 1 May‐30 June. Beta estimates, standard error (*SE*), and lower (LCI) and upper (UCI) 95% confidence intervals are presented.

FWC: female brown bears with cubs; M: adult male bear; S: subadult bears; SF: single female bear.

Day,

Wolf_day and

Wolf are the respective categorical reference on the intercept.

### 3.2 Spring (1 May–30 June)

The first six axes explained 75% of the marginality and were retained for the analysis (Figure 5b). Wolves and bears segregated from each other more than expected by chance (${\overset{¯}{S}}_{\mathrm{ij}}$ = 20.8%, *p* ≤ 0.01). Wolves segregated more than expected from all bear classes (from females with cubs, $S_{\mathrm{ij}}$ = 22.5%, *p* ≤ 0.01; from males, ${\overset{¯}{S}}_{\mathrm{ij}}$ = 19.5%, *p* ≤ 0.05; from single females, ${\overset{¯}{S}}_{\mathrm{ij}}$ = 21.3%, *p* ≤ 0.05; and from subadult bears, ${\overset{¯}{S}}_{\mathrm{ij}}$ = 23.3%, *p* ≤ 0.01). This segregation pattern was consistent during day and night (Table 1). Species and bear reproductive status were important variables to explain the variation in marginality scores, but only on the first axis of the K‐select (Table 2B). Consistently with the late‐winter period, wolves tended to select for moose occurrence, young forests, and rugged terrain more than bears did (Figure 5), as shown by the positive beta values for all bear classes (Table 3B, Axis 1).

On all other axes, both species tended to select habitat similarly, with time of day being the best variable to explain variation among individuals (for both species) in habitat selection. For example, wolves and bears showed a stronger selection for mid‐age forest, bogs, and areas farther from building and secondary roads during the day than at night (Figure 5b, Axis 4).

## 4 DISCUSSION

Our analyses of habitat selection within home ranges confirmed the hypothesis that Scandinavian wolves and bears segregated more than expected by chance during late winter and spring. Habitat segregation between wolves and bears was lower in late winter (${\overset{¯}{S}}_{\mathrm{ij}}$ = 14%) than in spring (${\overset{¯}{S}}_{\mathrm{ij}}$ = 20.8%), when segregation also involved more bear classes. In late winter, wolves segregated from male bears and female bears with cubs, whereas in spring, wolves segregated from all bear classes. Nevertheless, there were both differences and similarities in wolf and bear habitat selection, as shown by the selection on different axes of the K‐select analyses. The most distinctive pattern demonstrated that wolves selected for moose occurrence, young forests, and rugged terrain more than bears did, but both species showed general avoidance of human‐related infrastructure during daytime.

The stronger selection for moose by wolves than by bears was consistent throughout the two study periods and likely reflected differences in the requirements of an obligate carnivore compared to those of the omnivorous bear, whose diet is more diverse (Stenset et al., 2016). To account for marked seasonal differences in the behavior of wolves and bears and consider the phenology of the main prey and the progressive green‐up of vegetation, we divided our study of habitat selection into late winter and spring (Figure 3). Because ungulate calves are preyed upon efficiently by brown bears in spring, both in Eurasia (Swenson et al., 2007) and North America (Griffin et al., 2011), we expected wolf–bear habitat segregation to be lower during this period. However, segregation tended to be higher. Wolves segregated from all bear classes in the spring, compared to the late‐winter period. Wolves rear pups in spring, and all but one of our monitored wolves reproduced, which likely constrained their behavior, including their habitat selection. In addition, interspecific competition between wolves and bears occurs mostly at carcasses and may sometimes result in offspring death for both species (Ballard et al., 2003), which may also help to explain larger habitat segregation in spring, in addition to the fact that all bears are out of dens in spring (Figure 3).

Although the different diet requirements of wolves and bears may help to explain habitat segregation, their use of a common food resource could also lead to similarities in habitat selection. Wolves and bears actively prey on neonate moose in spring, and bears also feed on wolf‐killed moose (Milleret, 2011). Whereas neonate moose calves are small and are consumed quickly when preyed upon by either wolves or bears in spring, moose killed by wolves in late winter are larger, providing carcasses that take longer time to be consumed by wolves and bears (Wikenros, Sand, Ahlqvist, & Liberg, 2013). Thus, kleptoparasitism of wolf kills by bears in late winter, which is common in our study area (Milleret, 2011), could also explain the lower habitat segregation observed between wolves and bears in late winter than in spring.

We found that habitat selection of both species was affected similarly by time of the day. Wolves and bears avoided human‐related infrastructure during daytime, when outdoor human activities peak. Large carnivores generally avoid human‐dominated habitats and related features (Oriol‐Cotterill, Macdonald, Valeix, Ekwanga, & Frank, 2015), and Scandinavian bears and wolves are no exception (Ordiz et al., 2014, 2015; Zimmermann et al., 2014). Indeed, most mortality events are human‐related in Scandinavia for both bears (Bischof, Swenson, Yoccoz, Mysterud, & Gimenez, 2009) and wolves (Liberg et al., 2011; Milleret et al., 2017). Therefore, avoidance of human‐related habitats during daytime (a) reinforces previous findings of the strong effects that human activities have on large carnivore behavior in human‐dominated landscapes; and (b) may help to explain similarities in habitat selection, which could also be the result of wolves’ and bears’ predation/scavenging on the same prey. Similar findings have been reported for Eurasian lynx and wolverines in Scandinavia, where both species are also exposed to intensive human‐induced mortality and share the same prey species (Rauset, Mattisson, Andrén, Chapron, & Persson, 2012).

Although habitat overlap on some axes could suggest competition, partitioning on other axes may be sufficient to allow coexistence (Holt, 1987). Nevertheless, additional factors must be taken into account to interpret spatial interactions between sympatric species. This includes accounting for intraspecific factors that shape behavioral interactions among individuals (Grassel, Rachlow, & Williams, 2015). We defined two study periods that aligned with seasonal differences in the behavior of both wolves and bears (Figure 3) and explicitly took into account intra‐annual and daily individual variation in habitat selection (Uboni et al., 2015). Our K‐select analysis highlighted large individual variability in habitat selection that could not be explained solely by species and intraspecific characteristics. The limited sample size prevented us from having the statistical power required to distinguish wolf variability in habitat selection. Therefore, reproductive success, sex‐specific differences, and den location are factors that could explain the observed habitat selection variation among wolves. Individual variation in habitat selection and daily activity pattern have already been reported for bears (Gillies et al., 2006; Leclerc et al., 2015; Ordiz, Sæbø, Kindberg, Swenson, & Støen, 2017) and wolves (Hebblewhite & Merrill, 2008) and could be explained by differences in personality traits (Réale, Dingemanse, Kazem, & Wright, 2010). The large intraspecific variation found in our study may help wolves and bears to respond to intra‐ and interspecific competition and may promote coexistence (Vellend, 2006). Several lines of evidence suggest that intraspecific trait variation is important to promote species coexistence (Bolnick et al., 2011; Valladares, Bastias, Godoy, Granda, & Escudero, 2015).

The influence of seasonality on habitat selection deserves attention. At the intraspecific level, female bears with offspring segregate from other bears during the mating season in spring, but not during other seasons (Steyaert et al., 2013), and wolves also show seasonal variation in habitat selection (Uboni et al., 2015). Seasonality may also influence interspecific interactions, for example, with seasonal variation driven by changes in the predator's diet across the year (Saavedra, Rohr, Fortuna, Selva, & Bascompte, 2016). Bears are very efficient predators on neonatal moose calves during spring, but not on larger moose (Swenson et al., 2007). Later in the season, most bear populations rely on hard and soft mast (e.g. Naves et al., 2006). Therefore, the degree of trophic overlap between wolves and bears in summer and fall is certainly lower than in the spring. Accordingly, seasonality could change the degree of habitat segregation of wolves and bears we observed, which supports previous studies on the importance of seasonality to understand predator–prey interactions and predators’ co‐occurrence (Basille et al., 2013; Bastille‐Rousseau et al., 2016). Indeed, seasonal and even shorter (day–night) spatio‐temporal patterns may change the observed degree of segregation between sympatric species, which deserves further attention to understand the role of species interactions and how this affects their distribution pattern (Araújo & Rozenfeld, 2014).

Although wolves co‐occur with bears within similar habitat types at the landscape scale (May et al., 2008), wolf pairs avoid areas with high bear density when establishing territories (Ordiz et al., 2015). The spatial scale under consideration is crucial when studying biotic interactions (Araújo & Rozenfeld, 2014), and our study shows that habitat segregation between wolves and bears occurs at the home range scale. The patterns observed at different spatial scales (Ordiz et al., 2015 and this study) show that the result of biotic interactions might be visible at several scales and might act as a key mechanism allowing the coexistence between apex predators. Because most of the observed interactions between wolves and bears occur at carcasses (Ballard et al., 2003), fine‐scale movements around carcasses might be an additional mechanism used to reduce the risk of encounters and interactions, as recently described for other carnivores in Scandinavia (López‐Bao, Mattisson, Persson, Aronsson, & Andrén, 2016) and elsewhere. In Africa, for instance, habitat selection by cheetahs (*Acinonyx jubatus*) at the home range scale was similar to that of lions (*Panthera leo*) and spotted hyenas (*Crocuta crocuta*), but cheetahs avoided immediate risks by occurring farther from lions and hyenas than predicted by a random distribution (Broekhuis, Cozzi, Valeix, McNutt, & Macdonald, 2013).

Habitat segregation has been studied at different scales for many coexisting species, from spiders (Thompson, Ball, & Fitzgerald, 2015) to a variety of mammals, including ungulates (Darmon et al., 2012; Owen‐Smith, Martin, & Yoganand, 2015) and medium‐sized and large carnivores (Broekhuis et al., 2013; May et al., 2008; Pereira, Alves da Silva, Alves, Matos, & Fonseca, 2012). However, evidence of interspecific competition between sympatric large carnivores with fine‐scale data (e.g. GPS data) and at the population level is just beginning to be documented, in terms of both kill rates (Elbroch et al., 2015; Tallian et al., 2017) and habitat selection (Ordiz et al., 2015). Our study took advantage of the long‐term monitoring with GPS collars of brown bears and wolves in Scandinavia. Although the amount of data for individual wolves was much lower than for bears, our results showed that wolves and bears segregated within home ranges more than expected by chance, which might be a key mechanism allowing them to coexist. Our dataset included a large proportion of the bears inhabiting the area and at least one territorial leader wolf per existing pack. Whereas having GPS data from all bears and wolves in the area would have given us a more complete picture, this is hardly feasible in large carnivore studies and, beyond logistic, economic, and ethical considerations, we found no evidence that increasing the sample size would have caused dramatic changes in the observed patterns of habitat selection of either species. Therefore, we suggest that our results regarding habitat segregation are reliable.

Survival and partial recovery of wolves and other large carnivores in human‐dominated landscapes seems to be determined by an interaction between environmental and human factors, which reflects on the current distribution (Llaneza, López‐Bao, & Sazatornil, 2012) and genetic structure of wolf populations (Hulva et al., 2017). Nevertheless, to obtain a comprehensive understanding of the mechanisms facilitating coexistence among sympatric apex predators, it will also be important to understand how the habitat selection of each species is influenced by the relative density of the other species and by differences in availability of resources at large (Ordiz et al., 2015) and finer spatial scales (e.g. this study). In that sense, availability of resources used by one species, but not by the other, may also be important to understand interspecific differences in habitat selection. We focused our study in the part of the year when moose, particularly neonate calves, are important for both wolves and bears, but when the latter also relies on other resources rich in protein, for example, anthills (Stenset et al., 2016). Quantifying moose occurrence in such a vast area is challenging, and we used the best available data (pellet counts) to derive an index of moose occurrence. Although moose occurrence had a poor predictive power (low k‐fold cross‐validation), it was an important variable to explain bear and wolf habitat selection. Distribution and abundance of other resources (e.g. anthills), for instance, also may help to explain bear habitat selection and, potentially, differences with the habitat selection of wolves, which as obligate carnivores relied more specifically on moose. Therefore, it would be ideal to collect and include data on the availability of other resources in more holistic analyses to understand fully how coexisting species select habitat and share landscapes.

Disentangling the mechanisms driving interactions at different scales is also essential to understand how large carnivores’ coexistence affects lower trophic levels. For instance, there is substantial individual variation in the predatory behavior of brown bears, which has been reported in Scandinavia (Rauset, Kindberg, et al., 2012) and North America (Brockman et al., 2017). The estimated individual kill rates ranged from two to 15 moose calves per season among individual bears of the same sex and reproductive status in our study area (Rauset, Kindberg, et al., 2012). It is thus plausible that the habitat selection of highly predatory bears will share more similarity to wolf habitat selection than that of less predatory bears. This may help to explain the large individual variation in bear habitat selection that we found, and it deserves further research to quantify individual variation across years, for instance, because large individual variability in habitat selection of sympatric species may be a relevant mechanism promoting coexistence (Bolnick et al., 2011; Valladares et al., 2015).

## CONFLICT OF INTEREST

The authors declare no conflict of interests.

## AUTHORS’ CONTRIBUTIONS

CM, AO, and HS conceived the study; CM and AO wrote the manuscript; CM carried out statistical analysis, with contribution of GC; JM and AT helped building some habitat variables; BZ, CW, HPA, HS, JES, JK, JM, and PW coordinated and secured funding for the long‐term studies on bears and wolves; and all authors helped draft the manuscript and gave final approval for publication.

## DATA ACCESSIBILITY

Data are available on Dryad Digital Repository DOI: https://doi.org/10.5061/dryad.sc983fc.
