# Supplementary material for: Habitat segregation between brown bears and gray wolves in a human‐dominated landscape
Source: Ecol Evol. 2018 Nov 11;8(23):11450–66. doi: 10.1002/ece3.4572 (PMC6303696; doi:10.1002/ece3.4572)
Supplement: Supplementary file 1 [file ECE3-8-11450-s001.docx]

**Appendix 1**

**Table S1.** Reclassification of ‘Svenskt-Marktäckedata’ (SMD) land cover map (Lantmäteriet, Sweden; 25 x 25 m) used in the analysis of gray wolf and brown bear habitat selection in central Sweden, and percentage represented by each land cover class.

| **Original ID represented** | **Reclassification** | **Abbreviation** | **%** |
| --- | --- | --- | --- |
| 1. | Human settlements | Human | 0.8% |
| 2. | Agricultural fields | Agri | 1.5% |
| 3.1.1.1 & 3.1.1.2 | Deciduous forest | Deciduous | 1.4% |
| 3.1.2.1.1 | Coniferous forest on lichen | Coniferous | 7.1% |
| 3.1.2.2 | Coniferous forest on bog | Conifer_bogs | 1.9% |
| 3.1.2.1.2.1 | Midage coniferous forest | Middle | 27.5% |
| 3.1.2.1.2.2 & 3.1.2.3 & 3.3.2 | Mature coniferous forest | Mature | 23.8% |
| 3.1.3.1 & 3.1.3.2 | Mixed forest | Mixed | 2.5% |
| 3.2.4.1 & 3.2.4.3 | Young forest | Young | 10.9% |
| 3.2.4.2 | Clear cut | Clear_cut | 6% |
| 4. | Bogs | Bogs | 11.2% |
| 5. | Water | Water | 5.4% |

**Table S2.** AIC model selection results for the moose model (zero-inflated negative model) used to estimate spatial variation in moose occurrence in our study area in central Sweden. Description of the land use characteristics is provided in Table 1 of the main text. Dist1st and Dist2nd show Distance (km) to main and secondary roads, respectively. TRI7 is a terrain ruggedness index created using a 7 x 7moving window. Number of parameters (K), Akaike Information Criteria (AIC), ∆AIC, and AIC weight (AIC Wt) are presented.

| **Models** | **K** | **AIC** | **∆AIC** | **AIC Wt** |
| --- | --- | --- | --- | --- |
| Young + Bogs + Conifer_bogs + Dist2nd + Dist1st + DEM \|  Coniferous + Mid + Young + Mature + Mixed + Dist2nd + TRI7 + DEM | 17 | 7174.36 | 0 | 0.18 |
| Young + Bogs + Conifer_bogs + Dist2nd + Dist1st + DEM \|  Coniferous + Mid + Young + Mature + Dist2nd + TRI7 + DEM | 16 | 7174.494 | 0.134 | 0.17 |
| Young + Bogs + Conifer_bogs + Dist2nd + Dist1st + DEM \|  Coniferous + Mid + Young + Mature + Mixed + Clear_cut + Dist2nd + TRI7 + DEM | 18 | 7174.801 | 0.441 | 0.14 |
| Young + Mature + Bogs + Conifer_bogs + Dist2nd + Dist1st + DEM \|  Coniferous + Mid + Young + Mature + Mixed + Clear_cut + Dist2nd + TRI7 + DEM | 19 | 7175.372 | 1.012 | 0.11 |
| Young + Bogs + Conifer_bogs + Dist2nd + Dist1st + DEM \|  Mid + Young + Mature + Dist2nd + TRI7 + DEM | 15 | 7175.449 | 1.089 | 0.1 |
| Agri + Young + Bogs + Conifer_bogs + Dist2nd + Dist1st + DEM \|  Mid + Young + Mature + Dist2nd + TRI7 + DEM | 16 | 7176.084 | 1.724 | 0.08 |
| Deciduous + Young + Mature + Bogs + Conifer_bogs + Dist2nd + Dist1st + DEM \|  Deciduous + Coniferous + Mid + Young + Mature + Mixed + Clear_cut + Dist2nd + TRI7 + DEM | 21 | 7176.281 | 1.921 | 0.07 |
| Deciduous + Young + Mature + Bogs + Conifer_bogs + Dist2nd + Dist1st + DEM \|  Coniferous + Mid + Young + Mature + Mixed + Clear_cut + Dist2nd + TRI7 + DEM | 20 | 7177.239 | 2.879 | 0.04 |
| Deciduous + Young + Mature + Bogs + Conifer_bogs + Dist2nd + Dist1st + DEM \|  Deciduous + Coniferous + Mid + Young + Mature + Mixed + Clear_cut + Bogs + Dist2nd + TRI7 + DEM | 22 | 7177.341 | 2.981 | 0.04 |
| Deciduous + Young + Mature + Bogs + Conifer_bogs + Dist2nd + Dist1st + DEM \|  Deciduous + Coniferous + Mid + Young + Mature + Mixed + Clear_cut + Bogs + Conifer_bogs + Dist2nd + TRI7 + DEM | 23 | 7178.146 | 3.786 | 0.03 |
| Deciduous + Coniferous + Young + Mature + Bogs + Conifer_bogs + Dist2nd + Dist1st + DEM \| Deciduous + Coniferous + Mid + Young + Mature + Mixed + Clear_cut + Bogs + Conifer_bogs + Dist2nd + TRI7 + DEM | 24 | 7179.707 | 5.347 | 0.01 |
| Agri + Deciduous + Coniferous + Young + Mature + Mixed + Clear_cut + Bogs + Conifer_bogs + Dist2nd + Dist1st + DEM \|  Agri + Deciduous + Coniferous + Mid + Young + Mature + Mixed + Clear_cut + Bogs + Conifer_bogs + Dist2nd + TRI7 + DEM | 28 | 7179.944 | 5.584 | 0.01 |
| Deciduous + Coniferous + Young + Mature + Mixed + Bogs + Conifer_bogs + Dist2nd + Dist1st + DEM \|  Deciduous + Coniferous + Mid + Young + Mature + Mixed + Clear_cut + Bogs + Conifer_bogs + Dist2nd + TRI7 + DEM | 25 | 7181.662 | 7.302 | 0 |
| Agri + Deciduous + Coniferous + Young + Mature + Mixed + Clear_cut + Bogs + Conifer_bogs + Dist2nd + Dist1st + DEM \|  Agri + Deciduous + Coniferous + Mid + Young + Mature + Mixed + Clear_cut + Bogs + Conifer_bogs + Dist2nd + +Dist1st + TRI7 + DEM | 29 | 7181.867 | 7.507 | 0 |
| Deciduous + Coniferous + Young + Mature + Mixed + Clear_cut + Bogs + Conifer_bogs + Dist2nd + Dist1st + DEM \|  Deciduous + Coniferous + Mid + Young + Mature + Mixed + Clear_cut + Bogs + Conifer_bogs + Dist2nd + TRI7 + DEM | 26 | 7182.349 | 7.989 | 0 |
| Agri + Deciduous + Coniferous + Mid + Young + Mature + Mixed + Clear_cut + Bogs + Conifer_bogs + Dist2nd + Dist1st + DEM \|  Agri + Deciduous + Coniferous + Mid + Young + Mature + Mixed + Clear_cut + Bogs + Conifer_bogs + Dist2nd + +Dist1st + TRI7 + DEM | 30 | 7183.855 | 9.495 | 0 |
| Agri + Deciduous + Coniferous + Young + Mature + Mixed + Clear_cut + Bogs + Conifer_bogs + Dist2nd + Dist1st + DEM \|  Deciduous + Coniferous + Mid + Young + Mature + Mixed + Clear_cut + Bogs + Conifer_bogs + Dist2nd + TRI7 + DEM | 27 | 7184.018 | 9.658 | 0 |
| Agri + Deciduous + Coniferous + Mid + Young + Mature + Mixed + Clear_cut + Bogs + Conifer_bogs + Dist2nd + Dist1st + TRI7 + DEM +human\|  Agri + Deciduous + Coniferous + Mid + Young + Mature + Mixed + Clear_cut + Bogs + Conifer_bogs + Dist2nd + +Dist1st + TRI7 + DEM + human | 33 | 7188.157 | 13.80 | 0 |
| Null | 3 | 7567.932 | 393.572 | 0 |

**Table S3.** Parameter-averaged estimates of the best supported models (zero-inflated negative model) used to estimate spatial variation in moose occurrence in the study area in central Sweden. Description of the land use characteristics is provided in Table 1. Dist1st and Dist2nd show Distance (km) to main and secondary roads, respectively. TRI7 is a terrain ruggedness index created using a 7 x 7 moving window. All variables were scaled to facilitate interpretation.

| **Parameter** | **β** | **SE** |
| --- | --- | --- |
| **Binomial part (zero values)** |  |  |
| Intercept | -2.07 | 0.75 |
| Young forest | -0.57 | 0.15 |
| Mixed forest | -0.08 | 0.11 |
| Coniferous forest on bog | -0.17 | 0.13 |
| Dist2nd | 0.36 | 0.14 |
| TRI7 | -0.26 | 0.09 |
| Elevation | 0.01 | 0.01 |
| Mid age coniferous forest | -0.82 | 0.19 |
| Mature coniferous forest | -0.56 | 0.20 |
| Clear cut | -0.05 | 0.10 |
| Deciduous forest | -0.04 | 0.18 |
| **Negative binomial part (nonzero values)** |  |  |
| Intercept | -0.14 | 0.25 |
| Bogs | -0.19 | 0.09 |
| Young forest | 0.30 | 0.04 |
| Mature coniferous forest | -0.01 | 0.04 |
| Dist2nd | 0.27 | 0.07 |
| Dist1st | 0.19 | 0.05 |
| Elevation | 0.01 | 0.01 |
| Coniferous forest on bog | -0.18 | 0.07 |
| Agricultural fields | -0.01 | 0.03 |
| Deciduous forest | -0.01 | 0.02 |
|  |  |  |

**Figure S1.** Spatial prediction of moose occurrence in our study area in central Sweden based on pellet counts and zero-inflated negative models. High and low represents high to low number of pellets predicted (*Moose_pred*) by our best moose model. Red and blue dashed lines show the 95% MCP polygons for each individual brown bear and gray wolf, respectively.

**Figure A2**. Plot of the marginality vector of each individual brown bear (red) and gray wolf (blue) before a translation is applied to each vector so that they have the same origin. Marginality vectors represents available habitat conditions (start of the arrow) and average used conditions (end of the arrow) for each individual on the 2 first axis of the k-select during spring study period (1 May -30 June). This plot showed that the pattern of habitat selection (direction of marginality vector) seems independent of the available conditions within their home ranges.

**Table S4.** Summary of GPS locations from each individual gray wolf and brown bear included in the study in central Sweden to quantify habitat segregation between both species. For wolves, the name of the territory was used as the identification name. Sex of the individual for wolves, i.e. male (M) and female (F), and reproductive status of bears (females with cubs (FWC), single females (F), adult males (M), and sub-adult bears of both sexes (S) are noted. “Date.begin” and “Date.end” refer the date and time of the first and last position used for each individual, respectively. We show the data for the late-winter (1 March – 30 April) and the spring period of study (1 May – 30 June).

|  | **Late-winter Period** | | |  |  |
| --- | --- | --- | --- | --- | --- |
|  |  | **Wolf** |  |  |  |
| Territory | Sex | Date.begin | | Date.end | |
| Kukumaki | M | 3/5/2013 | 19:00:13 | 4/28/2013 | 23:00:44 |
| Kukumaki | M | 3/3/2014 | 1:00:50 | 4/25/2014 | 13:00:47 |
| Kukumaki | F | 3/4/2015 | 4:00:00 | 4/24/2015 | 5:00:00 |
| TandsjonA | M | 3/1/2012 | 0:00:44 | 4/30/2012 | 23:00:52 |
| TandsjonB | M | 3/19/2014 | 13:00:43 | 4/25/2014 | 23:00:39 |
| Tenskog | M | 3/1/2010 | 0:00:50 | 4/11/2010 | 3:00:56 |
| Tenskog | M | 3/14/2011 | 0:00:14 | 4/30/2011 | 23:00:42 |
|  |  |  |  |  |  |
|  |  | **Bear** |  |  |  |
| id | Sex | Date.begin | | Date.end | |
| W0104 | FWC | 4/1/2013 | 12:00:00 | 4/30/2013 | 23:30:00 |
| W0209 | FWC | 4/1/2010 | 2:31:00 | 4/30/2010 | 23:30:00 |
| W0217 | FWC | 4/1/2014 | 10:02:00 | 4/30/2014 | 23:00:00 |
| W0610 | SF | 4/1/2014 | 0:00:00 | 4/30/2014 | 23:00:00 |
| W0620 | FWC | 4/1/2014 | 0:00:00 | 4/30/2014 | 23:00:00 |
| W0625 | M | 4/1/2010 | 9:33:00 | 4/30/2010 | 23:30:00 |
| W0625 | M | 4/11/2011 | 11:30:00 | 4/30/2011 | 23:30:00 |
| W0625 | M | 4/18/2013 | 16:30:00 | 4/30/2013 | 23:30:00 |
| W0703 | SF | 4/11/2011 | 13:34:00 | 4/30/2011 | 23:30:00 |
| W0716 | FWC | 4/1/2013 | 0:31:00 | 4/30/2013 | 23:30:00 |
| W0719 | M | 4/1/2011 | 0:01:00 | 4/30/2011 | 23:30:00 |
| W0720 | FWC | 4/4/2011 | 10:33:00 | 4/30/2011 | 23:30:00 |
| W0802 | M | 4/1/2011 | 2:01:00 | 4/30/2011 | 23:31:00 |
| W0805 | M | 4/1/2011 | 0:00:00 | 4/30/2011 | 23:30:00 |
| W0818 | SF | 4/2/2014 | 0:33:00 | 4/30/2014 | 23:30:00 |
| W0825 | SF | 4/2/2011 | 11:31:00 | 4/30/2011 | 23:30:00 |
| W0825 | SF | 4/1/2014 | 0:00:00 | 4/30/2014 | 23:00:00 |
| W1001 | M | 4/12/2010 | 13:29:00 | 4/30/2010 | 23:30:00 |
| W1001 | M | 4/1/2011 | 0:02:00 | 4/30/2011 | 23:30:00 |
| W1017 | SF | 4/1/2014 | 1:01:00 | 4/30/2014 | 23:02:00 |
| W1020 | M | 4/6/2011 | 14:59:00 | 4/30/2011 | 23:30:00 |
| W1110 | Sub | 4/1/2012 | 9:32:00 | 4/30/2012 | 23:30:00 |
| W1110 | SF | 4/5/2014 | 13:02:00 | 4/30/2014 | 23:00:00 |
| W1203 | SF | 4/1/2014 | 0:02:00 | 4/30/2014 | 23:30:00 |
| W1204 | Sub | 4/1/2013 | 0:00:00 | 4/30/2013 | 23:30:00 |
| W1204 | M | 4/1/2014 | 0:00:00 | 4/30/2014 | 23:00:00 |
| W1205 | Sub | 4/1/2014 | 0:01:00 | 4/30/2014 | 23:00:00 |
| W1206 | Sub | 4/1/2014 | 1:02:00 | 4/30/2014 | 23:00:00 |
| W1206 | SF | 4/1/2015 | 12:00:00 | 4/30/2015 | 23:00:00 |
| W1209 | Sub | 4/1/2014 | 0:01:00 | 4/30/2014 | 23:02:00 |
| W1211 | M | 4/19/2013 | 9:30:00 | 4/30/2013 | 23:30:00 |
| W1211 | M | 4/1/2014 | 0:01:00 | 4/30/2014 | 23:00:00 |
| W1211 | M | 4/1/2015 | 0:00:00 | 4/30/2015 | 23:00:00 |
| W1303 | Sub | 4/1/2014 | 0:00:00 | 4/30/2014 | 23:00:00 |
| W1306 | Sub | 4/1/2014 | 0:00:00 | 4/30/2014 | 23:00:00 |
| W1314 | M | 4/1/2014 | 13:00:00 | 4/30/2014 | 23:00:00 |
| W1314 | M | 4/1/2015 | 0:01:00 | 4/30/2015 | 23:01:00 |
| W1416 | M | 4/1/2015 | 0:00:00 | 4/30/2015 | 23:01:00 |
| W9301 | M | 4/4/2010 | 19:00:00 | 4/30/2010 | 23:30:00 |
| W9403 | FWC | 4/3/2010 | 9:33:00 | 4/30/2010 | 23:30:00 |

|  | **Spring Period** | | |  |  |
| --- | --- | --- | --- | --- | --- |
|  |  | Wolf |  |  |  |
| Territory | Sex | date.begin | | date.end | |
| Kukumaki | M | 5/19/2014 | 1:00:46 | 6/22/2014 | 23:00:48 |
| Kukumaki | F | 5/18/2015 | 1:01:00 | 6/29/2015 | 16:00:00 |
| Tandsjon | M | 5/19/2014 | 1:02:08 | 6/21/2014 | 11:00:44 |
| Tandsjon | M | 5/1/2012 | 0:01:19 | 5/14/2012 | 0:00:48 |
| Tenskog | M | 5/30/2011 | 0:00:13 | 6/26/2011 | 23:00:43 |
| Tenskog | M | 5/1/2011 | 0:00:48 | 5/15/2011 | 23:00:41 |
|  |  |  |  |  |  |
|  |  | Bear |  |  |  |
| id | sex | date.begin | | date.end | |
| W0104 | FWC | 5/1/2013 | 0:00:00 | 5/31/2013 | 23:30:00 |
| W0104 | SF | 6/1/2013 | 0:00:00 | 6/30/2013 | 23:30:00 |
| W0104 | FWC | 5/1/2015 | 0:00:00 | 6/30/2015 | 9:01:00 |
| W0209 | FWC | 5/1/2010 | 0:01:00 | 6/23/2010 | 14:30:00 |
| W0217 | FWC | 5/1/2014 | 2:02:00 | 6/5/2014 | 21:01:00 |
| W0425 | FWC | 5/1/2010 | 0:01:00 | 6/30/2010 | 23:30:00 |
| W0425 | FWC | 5/1/2011 | 0:02:00 | 6/30/2011 | 23:30:00 |
| W0425 | SF | 5/1/2014 | 0:00:00 | 6/30/2014 | 23:00:00 |
| W0517 | SF | 5/1/2012 | 2:00:00 | 6/30/2012 | 23:00:00 |
| W0605 | FWC | 5/1/2014 | 0:00:00 | 6/30/2014 | 23:02:00 |
| W0610 | SF | 5/1/2011 | 0:00:00 | 6/30/2011 | 23:30:00 |
| W0610 | SF | 5/1/2014 | 0:00:00 | 6/30/2014 | 23:00:00 |
| W0611 | SF | 5/1/2010 | 0:00:00 | 6/30/2010 | 23:30:00 |
| W0611 | SF | 5/1/2011 | 0:00:00 | 6/30/2011 | 23:30:00 |
| W0620 | FWC | 5/1/2014 | 0:00:00 | 6/30/2014 | 23:00:00 |
| W0625 | M | 5/1/2010 | 0:00:00 | 6/30/2010 | 23:30:00 |
| W0625 | M | 5/1/2011 | 0:00:00 | 6/30/2011 | 23:30:00 |
| W0625 | M | 5/1/2013 | 0:00:00 | 6/10/2013 | 23:30:00 |
| W0625 | M | 5/1/2014 | 0:00:00 | 6/30/2014 | 23:00:00 |
| W0703 | SF | 5/1/2011 | 0:01:00 | 6/30/2011 | 23:30:00 |
| W0716 | FWC | 5/1/2013 | 0:00:00 | 6/30/2013 | 22:31:00 |
| W0716 | FWC | 5/1/2014 | 0:00:00 | 6/30/2014 | 23:01:00 |
| W0719 | M | 5/1/2011 | 0:00:00 | 6/30/2011 | 23:30:00 |
| W0719 | M | 5/1/2014 | 0:00:00 | 6/30/2014 | 23:00:00 |
| W0720 | FWC | 5/1/2011 | 0:00:00 | 6/30/2011 | 23:30:00 |
| W0720 | FWC | 5/1/2014 | 0:00:00 | 6/30/2014 | 23:00:00 |
| W0802 | M | 5/1/2011 | 0:00:00 | 6/30/2011 | 23:30:00 |
| W0805 | M | 5/1/2010 | 0:00:00 | 6/30/2010 | 23:30:00 |
| W0805 | M | 5/1/2011 | 0:00:00 | 6/30/2011 | 23:31:00 |
| W0806 | SF | 5/1/2014 | 0:00:00 | 6/30/2014 | 23:00:00 |
| W0818 | SF | 5/1/2014 | 0:00:00 | 6/30/2014 | 21:01:00 |
| W0825 | SF | 5/1/2011 | 0:00:00 | 6/30/2011 | 23:30:00 |
| W0825 | SF | 5/1/2014 | 0:02:00 | 6/30/2014 | 23:01:00 |
| W0910 | M | 5/1/2014 | 1:00:00 | 6/30/2014 | 23:01:00 |
| W1001 | M | 5/1/2010 | 0:00:00 | 6/30/2010 | 23:30:00 |
| W1001 | M | 5/1/2011 | 0:00:00 | 6/30/2011 | 23:30:00 |
| W1011 | SF | 5/1/2014 | 0:00:00 | 6/30/2014 | 23:00:00 |
| W1017 | Sub | 5/1/2011 | 0:32:00 | 6/30/2011 | 23:30:00 |
| W1020 | M | 5/1/2011 | 0:00:00 | 6/30/2011 | 23:30:00 |
| W1105 | SF | 5/1/2014 | 0:00:00 | 6/30/2014 | 23:01:00 |
| W1110 | Sub | 5/1/2012 | 0:00:00 | 6/30/2012 | 23:30:00 |
| W1110 | SF | 5/1/2014 | 0:00:00 | 6/30/2014 | 23:01:00 |
| W1203 | SF | 5/1/2014 | 0:00:00 | 6/30/2014 | 21:00:00 |
| W1204 | Sub | 5/1/2013 | 0:00:00 | 6/30/2013 | 23:30:00 |
| W1204 | M | 5/1/2014 | 0:01:00 | 6/30/2014 | 23:00:00 |
| W1205 | Sub | 5/1/2013 | 0:00:00 | 6/30/2013 | 23:31:00 |
| W1205 | Sub | 5/1/2014 | 1:00:00 | 6/30/2014 | 23:00:00 |
| W1205 | SF | 5/1/2015 | 0:00:00 | 6/30/2015 | 23:00:00 |
| W1206 | Sub | 5/1/2014 | 0:00:00 | 6/30/2014 | 23:00:00 |
| W1206 | SF | 5/1/2015 | 0:00:00 | 6/29/2015 | 12:00:00 |
| W1209 | Sub | 5/1/2014 | 0:02:00 | 6/30/2014 | 23:01:00 |
| W1210 | M | 5/1/2014 | 0:00:00 | 6/30/2014 | 23:00:00 |
| W1211 | M | 5/1/2013 | 0:00:00 | 6/30/2013 | 23:30:00 |
| W1211 | M | 5/1/2014 | 0:00:00 | 6/30/2014 | 22:00:00 |
| W1211 | M | 5/1/2015 | 0:00:00 | 6/29/2015 | 4:01:00 |
| W1301 | Sub | 5/1/2014 | 0:00:00 | 6/6/2014 | 6:00:00 |
| W1302 | Sub | 5/1/2014 | 0:00:00 | 6/30/2014 | 22:00:00 |
| W1303 | Sub | 5/1/2014 | 0:02:00 | 6/30/2014 | 23:00:00 |
| W1304 | Sub | 5/1/2014 | 0:00:00 | 6/30/2014 | 23:02:00 |
| W1305 | Sub | 5/1/2014 | 0:02:00 | 6/30/2014 | 23:00:00 |
| W1306 | Sub | 5/1/2014 | 1:00:00 | 6/20/2014 | 2:00:00 |
| W1307 | Sub | 5/1/2014 | 0:00:00 | 6/30/2014 | 23:00:00 |
| W1308 | Sub | 5/1/2014 | 0:00:00 | 6/30/2014 | 23:00:00 |
| W1312 | M | 5/1/2014 | 0:00:00 | 6/30/2014 | 23:01:00 |
| W1314 | M | 5/15/2013 | 14:30:00 | 6/27/2013 | 2:00:00 |
| W1314 | M | 5/1/2014 | 0:00:00 | 6/25/2014 | 20:59:00 |
| W1314 | M | 5/1/2015 | 0:00:00 | 6/30/2015 | 23:00:00 |
| W1316 | Sub | 5/1/2014 | 0:00:00 | 6/30/2014 | 23:02:00 |
| W1317 | Sub | 5/1/2014 | 0:00:00 | 6/30/2014 | 23:00:00 |
| W1319 | FWC | 5/1/2014 | 0:00:00 | 6/30/2014 | 23:00:00 |
| W1407 | Sub | 5/1/2014 | 0:00:00 | 6/30/2014 | 23:00:00 |
| W1408 | Sub | 5/1/2014 | 0:00:00 | 6/30/2014 | 23:00:00 |
| W1416 | M | 6/4/2014 | 21:00:00 | 6/30/2014 | 22:00:00 |
| W1416 | M | 5/1/2015 | 0:00:00 | 6/30/2015 | 23:00:00 |
| W1417 | SF | 6/5/2014 | 14:00:00 | 6/30/2014 | 23:01:00 |
| W1505 | SF | 5/12/2015 | 13:00:00 | 6/30/2015 | 23:00:00 |
| W9301 | M | 5/1/2010 | 0:00:00 | 6/30/2010 | 23:30:00 |
| W9403 | FWC | 5/1/2010 | 0:00:00 | 6/30/2010 | 23:30:00 |
| W9403 | FWC | 5/1/2014 | 0:02:00 | 6/30/2014 | 23:30:00 |


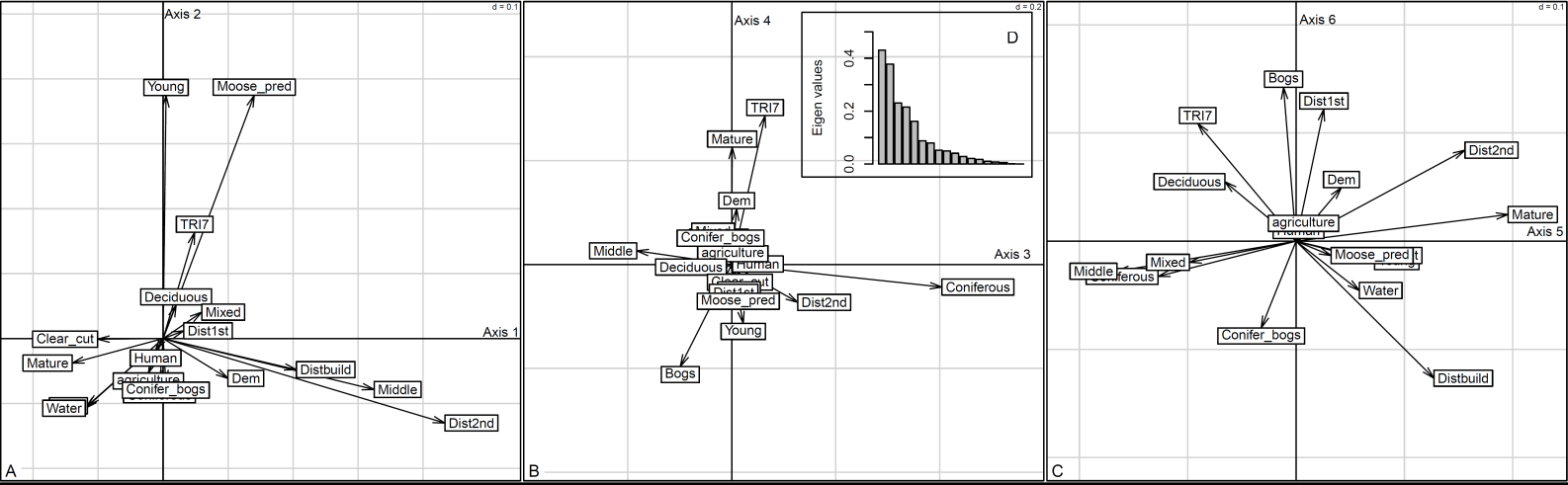

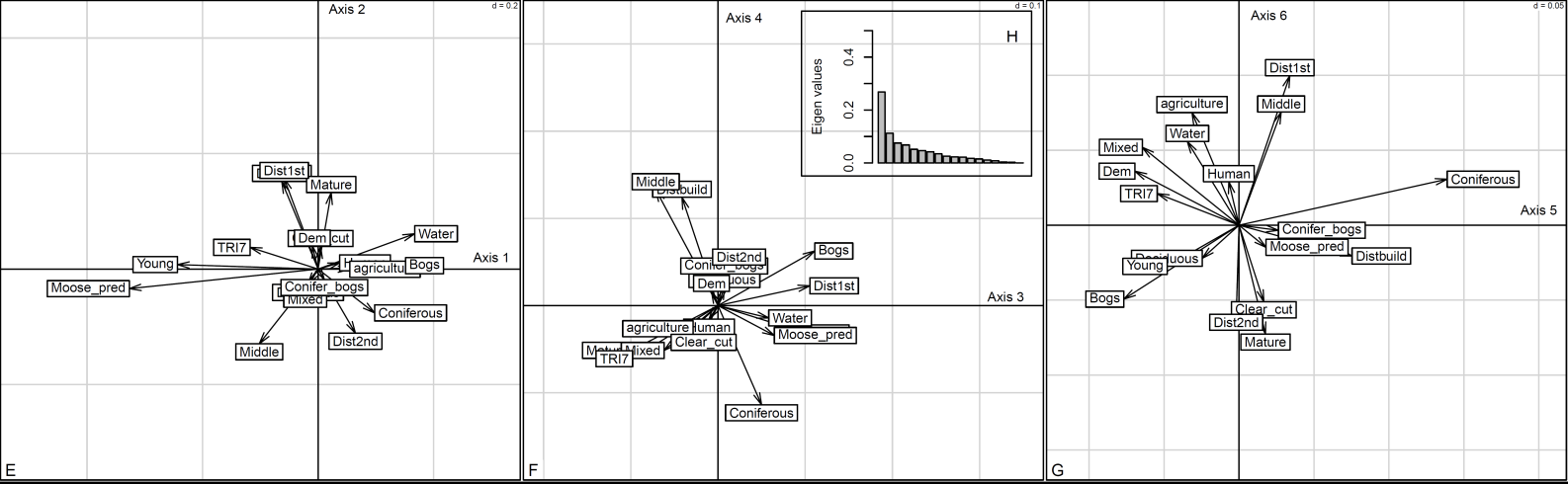


**Figure S3.** Variable loadings on the factorial axes in our study on gray wolf and brown bear habitat selection and segregation in central Sweden. Plots in the top panel (A-D) correspond to late-winter period (1 March - 30 April) and plots in the bottom panel (E-H) correspond to the spring period (1 May - 30 June). The 3 graphics in each panel show axes 1-2, 3-4, and 5-6, respectively. Graphics D and H shows the bar chart of the K-select eigenvalues, measuring the mean marginality explained by each factorial axis, for the late-winter and spring period, respectively.


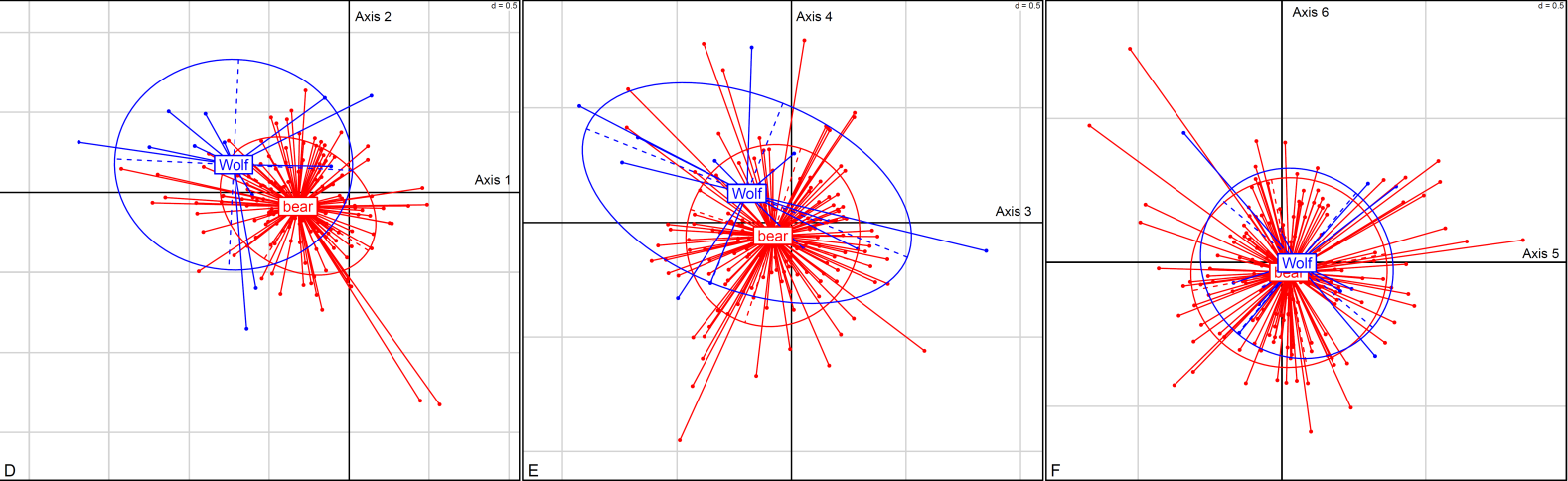

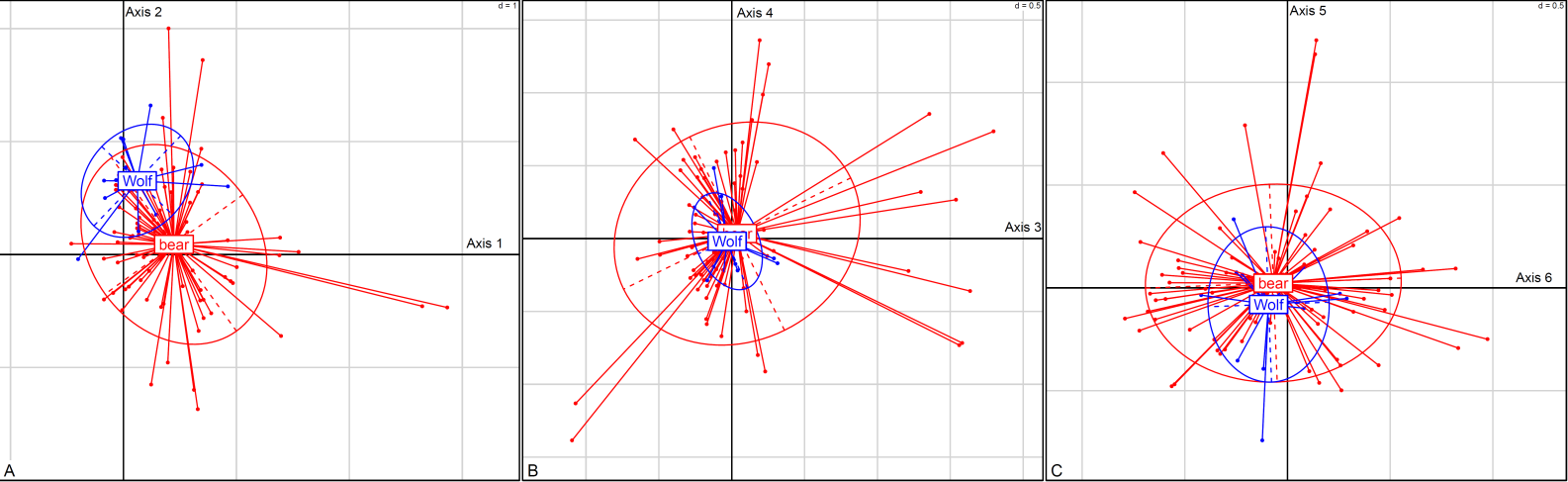


**Figure S4** Marginality scores projected on the K-select analysis of each individual gray wolf (blue) and brown bear (red) in central Sweden, after re-centering on each individual home range composition. Each individual-year is represented by 2 points, one for diurnal habitat selection and one for nocturnal habitat selection. Differences in marginality vectors were merged and ellipsed by species. Plots in the top panel (A-C) correspond to the late-winter study period (1 March - 30 April) and plots in the bottom panel (D-F) correspond to the spring study period (1 May -30 June). The 3 graphics in each panel show axes 1-2, 3-4, and 5-6, respectively.
